# Metabolism of 4-chloro-2-nitrophenol in a Gram-positive bacterium, *Exiguobacterium* sp. PMA

**DOI:** 10.1186/1475-2859-11-150

**Published:** 2012-11-21

**Authors:** Pankaj Kumar Arora, Ashutosh Sharma, Richa Mehta, Belle Damodara Shenoy, Alok Srivastava, Vijay Pal Singh

**Affiliations:** 1 Microbial Type Culture Collection (MTCC) and Gene Bank, CSIR-Institute of Microbial Technology, Sec-39A, Chandigarh 1600036, India; 2 Department of Plant Sciences, School of Life Sciences, University of Hyderabad, P.O Central University, Hyderabad, 500 046, India; 3 Escuela de Ingenieria en Alimentos, Biotecnologia y Agronomia, Instituto Tecnologico y de Estudios Superiores de Monterrey, Epigmenio Gonzalez 500, Col. San Pablo, Queretaro, Mexico; 4 Centro de Investigación en Biotecnología (CEIB), La Universidad Autonoma del Estado de Morelos (UAEM), Av. Universidad 1001, Col. Chamilpa, Cuernavaca, Morelos, 62209, Mexico; 5 Department of Plant Science, Faculty of Applied Sciences, MJP Rohilkhand University, Bareilly, 243006, India

**Keywords:** 4-Chloro-2-nitrophenol, 4-Chloro-2-aminophenol, 2-aminophenol, Biodegradation, Bioremediation, Soil microcosm

## Abstract

**Background:**

Chloronitrophenols (CNPs) are widely used in the synthesis of dyes, drugs and pesticides, and constitute a major group of environmental pollutants. 4-Chloro-2-nitrophenol (4C2NP) is an isomer of CNPs that has been detected in various industrial effluents. A number of physicochemical methods have been used for treatment of wastewater containing 4C2NP. These methods are not as effective as microbial degradation, however.

**Results:**

A 4C2NP-degrading bacterium, *Exiguobacterium* sp. PMA, which uses 4C2NP as the sole carbon and energy source was isolated from a chemically-contaminated site in India. *Exiguobacterium* sp. PMA degraded 4C2NP with the release of stoichiometeric amounts of chloride and ammonium ions. The effects of different substrate concentrations and various inoculum sizes on degradation of 4C2NP were investigated. *Exiguobacterium* sp. PMA degraded 4C2NP up to a concentration of 0.6 mM. High performance liquid chromatography and gas chromatography–mass spectrometry identified 4-chloro-2-aminophenol (4C2AP) and 2-aminophenol (2AP) as possible metabolites of the 4C2NP degradation pathway. The crude extract of 4C2NP-induced PMA cells contained enzymatic activity for 4C2NP reductase and 4C2AP dehalogenase, suggesting the involvement of these enzymes in the degradation of 4C2NP. Microcosm studies using sterile and non-sterile soils spiked with 4C2NP were carried out to monitor the bioremediation potential of *Exiguobacterium* sp. PMA. The bioremediation of 4C2NP by *Exiguobacterium* sp. PMA was faster in non-sterilized soil than sterilized soil.

**Conclusions:**

Our studies indicate that *Exiguobacterium* sp. PMA may be useful for the bioremediation of 4C2NP-contaminated sites. This is the first report of (i) the formation of 2AP in the 4C2NP degradation pathway by any bacterium and (iii) the bioremediation of 4C2NP by any bacterium.

## Background

Chloronitrophenols (CNPs) are widely used in the synthesis of dyes, drugs and pesticides, and constitute a major group of environmental pollutants [1]. 4-Chloro-2-nitrophenol (4C2NP) and 2-chloro-4-nitrophenol (2C4NP) are the two most common isomers of CNP that have been detected in various industrial effluents [1,2]. A number of physicochemical methods have been used for treatment of wastewater containing CNPs [1]. These methods are not as effective as microbial degradation, however [1-5].

Microbial degradation of CNPs may be initiated by either an oxidative or a reductive process. In the oxidative process, an oxygenase attacks the benzene ring by removing nitrite ion, and degradation proceeds further via chloride release and ring cleavage [6,7]. In the reductive process, degradation of CNPs proceeds by one of the following mechanisms: (i) the reduction of the nitro group into hydroxylamine [8] or the amino group [2]; or (ii) by the reductive removal of chloride ion [9].

In this study, we have selected 4C2NP as the model compound for the study of the degradation of CNPs. Few studies on the bacterial degradation of 4C2NP have been reported. Beunink and Rehm [10] reported the degradation of 4C2NP by a co-culture of *Enterobacter cloacae* and an *Alcaligenes* sp. TK-2. Bruhn et al. [11] constructed a genetically engineered bacterium, *Pseudomonas* sp. N31 that mineralized 2C4NP by the removal of chloride and nitrite ions. Arora and Jain [2] reported detoxification of 4C2NP by reduction and subsequent acetylation.

4C2NP is structurally very similar to 2C4NP, with the same molecular formula (C_6_H_4_NO_**3**_Cl) and molecular weight (173.5). The difference is in the change of the positions of the chloro and nitro groups at the benzene ring. There are several bacteria that utilize 2C4NP as their sole carbon and energy sources, including *Arthrobacter nitrophenolicus* SJCon [6,12], *Burkholderia* sp. SJ98 [9], *Burkholderia* sp. RKJ 800 [13] and *Rhodococcus imtechensis* RKJ300 [7]. All of these bacteria degrade 2C4NP, but were not able to degrade 4C2NP. This is due to the fact that enzymes that act at the *para* positions can not act at the *ortho* or *meta* positions, and vice versa [1,13]. The aromatic compounds that have nitro groups at *ortho* or *meta* positions are considered to be more resistant to microbial attack than the compounds that have nitro groups at *para* positions [1,13]. Therefore, 4C2NP is more recalcitrant than 2C4NP.

The present communication describes: (i) the isolation of an efficient 4C2NP mineralizing bacterium, *Exiguobacterium* sp. PMA; (ii) the metabolic pathway of degradation of 4C2NP by *Exiguobacterium* sp. PMA and (iii) bioremediation of 4C2NP in the soil using *Exiguobacterium* sp. PMA.

## Results

### Isolation and Identification of 4C2NP degrading bacterium

A 4C2NP degrading bacterial strain PMA was isolated from a chemically-contaminated site of India by enrichment method that utilized 4C2NP as the sole carbon and energy source. Strain PMA was identified as *Exiguobacterium* sp. on the basis of the 16S rRNA gene sequence analysis. The 16S rRNA gene sequence of *Exiguobacterium* sp. PMA was deposited in the GenBank under the accession number JQ182409. *Exiguobacterium* sp. PMA was screened for its ability to degrade other nitroaromatic compounds. It was observed that *Exiguobacterium* sp. PMA degraded 4C2NP, 2-nitrophenol (2NP), 4-chloro-2-aminophenol (4C2AP) and 2-aminophenol (2-AP) but unable to degrade 2-chloro-4-nitrophenol (2C4NP), 2-methyl-4-nitrophenol (2Me4NP), 3-methyl-4-nitrophenol (3Me4NP) and 4-nitrophenol (4NP) (Table 1).

**Table 1:** **Screening of *Exiguobacterium *sp. PMA for its ability to utilize various nitroaromatic compounds as the sole carbon and energy sources**

**Name of Compound(s)**	**Utilization of compounds as carbon and energy sources by *Exiguobacterium *sp**. **PMA**	
	**Decolourization of minimal agar plates containing 0.3 mM test compound(s) as sole carbon and energy sources**	**Growth of strain PMA on minimal agar plates containing 0.3 mM test compound(s) as sole carbon and engery sources**
4-Chloro-2-Nitrophenol (4C2NP)	Yes	Yes
4-Chloro-2-Aminophenol (4C2AP)	Yes	Yes
2-Chloro-4-Nitrophenol (2C4NP)	No	No
2-Nitrophenol (2NP)	Yes	Yes
4-Nitrophenol (4NP)	No	No
2-Methyl-4-Nitrophenol (2Me4NP)	No	No
3-Methyl-4-Nitrophenol (3Me4NP)	No	No
2-Aminophenol (2AP)	Yes	Yes

### Growth and degradation studies

**Figure 1:**
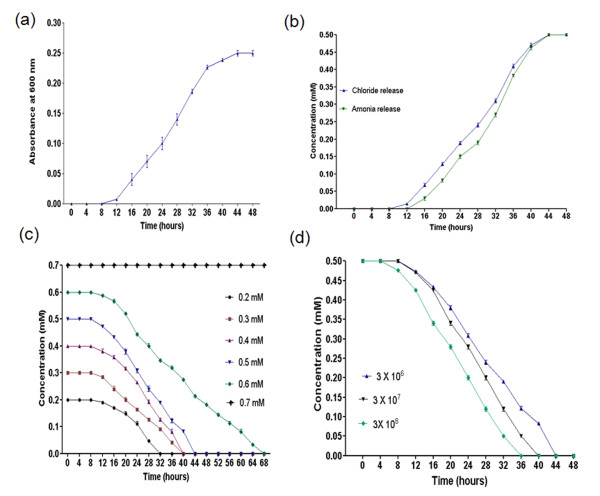
**Growth and Degradation Studies.** (**a**) Utilization of 4C2NP as a sole source of carbon and energy by *Exiguobacterium* sp. PMA. (**b**) Estimation of chloride and ammonia releases from 4C2NP by *Exiguobacterium* sp. PMA. (**c**) Effect of various substrate concentrations on degradation of 4C2NP by *Exiguobacterium* sp. PMA. (**d**) Effects on different inaculum sizes on degradation of 4C2NP.

When *Exiguobacterium* sp. PMA was grown on minimal media containing 0.5 mM 4C2NP as sole of carbon and energy source, the yellow color of 4C2NP changed to colorless indicating its utilization by *Exiguobacterium* sp. PMA. The utilization of 4C2NP was accompanied by concomitant increase in cell growth that reached a maximum growth equivalent to OD_600_ of 0.250 (Figure 1a). *Exiguobacterium* sp. PMA degraded 4C2NP completely within 44 hours and the stoichiometric amounts of chloride and ammonium ions were detected during the degradation of 4C2NP (Figure 1b).

### Effects of substrate concentrations on degradation

No degradation was observed when *Exiguobacterium* sp. PMA was grown on minimal medium containing 0.7 mM 4C2NP. Degradation was observed when the range of the 4C2NP concentration was from 0.1 mM to 0.6 mM (Figure 1c). The optimum concentration for degradation of 4C2NP by *Exiguobacterium* sp. PMA was determined as 0.5 mM on the basis of highest growth at this concentration. This concentration was selected for whole study.

### Effect of different inoculum sizes on 4C2NP degradation

4C2NP was degraded by *Exiguobacterium* sp. PMA during all initial cell densities tested. In culture inoculated with highest cell densities, the degradation of 4C2NP was faster with compared to cultures having lower inoculum densities (Figure 1d).

### Identification of Metabolites

**Figure 2:**
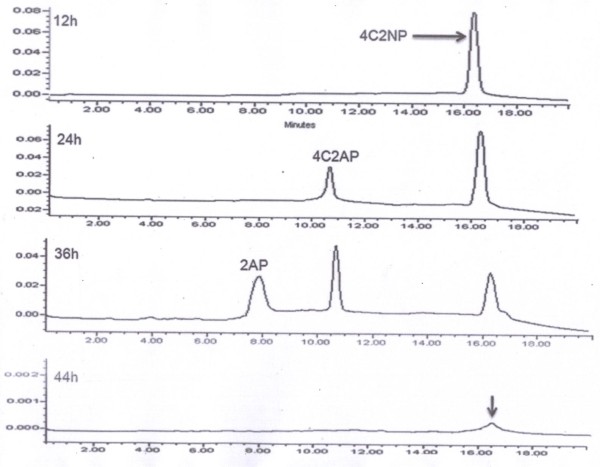
**HPLC elution profile of samples of degradation of 4C2NP by *Exiguobacterium *sp. PMA.** HPLC confirmed complete depletion of 4C2NP by *Exiguobacterium* sp. PMA within 44 h. Metabolite I was detected in sample of 24 and 36 h whereas metabolite 11 was detected only in the sample of 36 h.

High Performance Liquid Chromatography (HPLC) and Gas chromatography–mass spectrometry (GC-MS) studies were carried out to elucidate metabolic pathway of 4C2NP in *Exiguobacterium* sp. PMA. HPLC confirmed complete depletion of 4C2NP by *Exiguobacterium* sp. PMA within 44 h (Figure 2). In the 12 h sample, only parent compound was detected. The metabolite I was detected in the sample of 24 h and 36 h whereas the metabolite II was detected only in the 36 h sample. In the sample of 44 h, neither parent compound nor metabolite was detected. The retention time of 4C2NP, metabolite 1 and metabolite II were 16.4 min, 10.6 min and 7.7 min, respectively. The retention times of metabolite I and II were exactly match with that of authentic 4-chloro-2-aminophenol (4C2AP) and 2-aminophenol (2AP).

**Figure 3:**
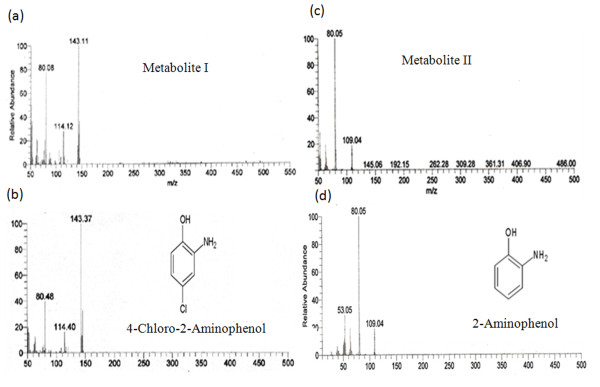
**Mass spectra of metabolites and authenetic standards.** (**a**) Metabolite I, (**b**) 4-Chloro-2-aminophenol (4C2AP), (**c**) Metabolite II, and (**d**) 2-Aminophenol (2AP).

To identify both of the metabolites, GC-MS was carried out. The mass spectrum of metabolite I was observed at 143 m/z that was identical to authentic 4C2AP (Figure 3a and 3b). The mass spectrum of metabolite II was observed at 109 m/z that was identical to authentic 2AP (Figure 3c and 3d). On the basis of GC-MS, metabolite 1 and 11 were identified as 4C2AP and 2AP, respectively.

### Enzyme assays

In the crude extract of the 4C2NP induced cells of *Exiguobacterium* sp. PMA, we have detected enzyme activities of 4C2NP nitroreductase and 4C2AP dehalogenase. 4C2NP reductase catalyzed the conversion of 4C2NP into 4C2AP by the reduction of nitro group of 4C2NP into amino group. The activity of 4C2NP reductase was determined by detection of 4C2AP by GC-MS after the incubation the reaction mix at 30°C for 10 minutes. No 4C2AP was detected in the control.

Another enzyme, 4C2AP dehalogenase catalyzed the conversion of 4C2AP to 2AP with removal of chloride ions. The stoichiometric amounts of chloride ions were detected during the enzyme assay. 2AP was detected as a product of activity of 4C2AP dehalogenase. In the control, neither chloride release was observed nor 2AP was detected.

### Ring cleavage inhibition studies

**Figure 4:**
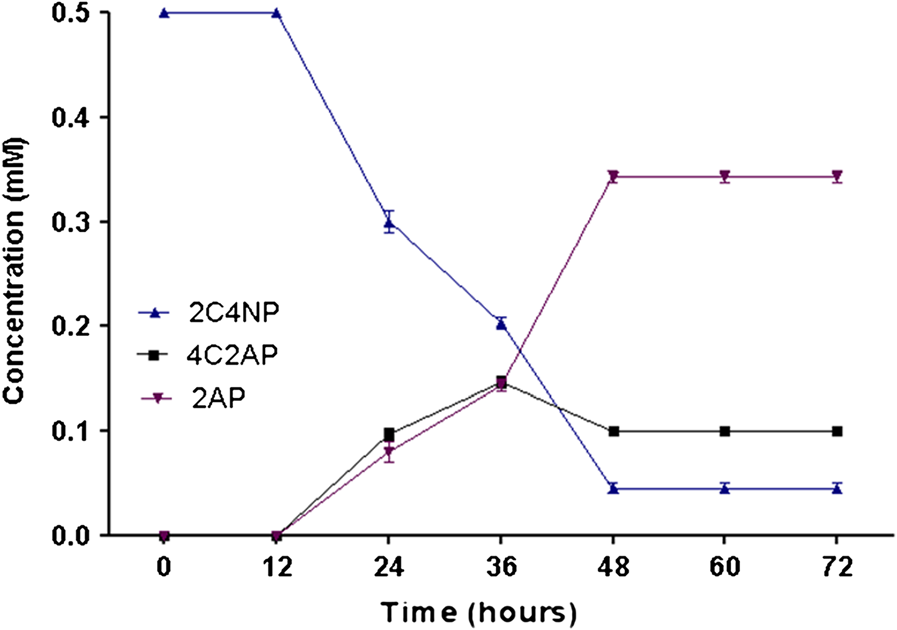
**Ring cleavage inhibition studies.** Dipyridyl inhibits the ring cleavege to 2-aminophenol and as a result, 2-aminophenol and 4-chloro-2-aminophenol were accumulated in the media in significant amounts.

The results of inhibition studies showed the accumulation of 2AP (0.35 mM) and 4C2AP (0.1 mM) in the medium (Figure 4). Dipyridyl prevents the ring cleavage of 2AP by chelating the ferrous ions required by dioxygenase to cleave the aromatic ring. Due to blockage of the ring cleavage of 2AP, 2AP and 4C2AP were accumulated in the media. These results showed that 4C2AP and 2AP both are major metabolites of degradation pathway of 4C2NP.

### Microcosm studies

In order to determine the capability of *Exiguobacterium* sp. PMA to degrade 4C2NP in the soil, we performed microcosm studies using both sterile and non-sterile soils under optimized conditions. The optimized parameters were as follows: inoculum size 2 × 10^7^ CFU g^−1^ soil, pH 7.5, temperature 30°C, and substrate concentration 100 ppm of 4C2NP.

**Figure 5:**
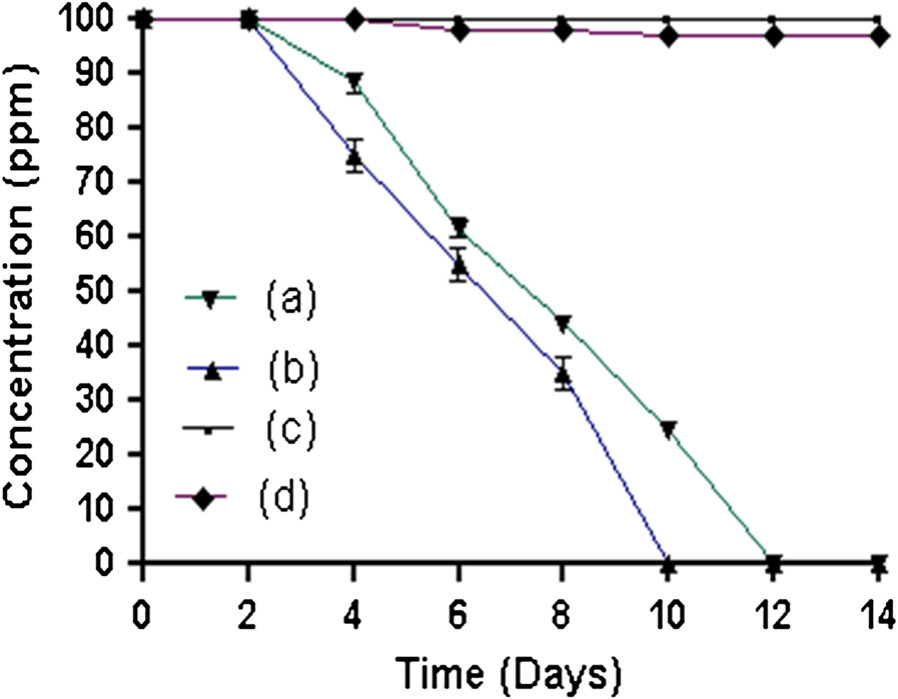
**Microcosm studies.** (**a**) Degradation of 4C2NP in sterile soil by *Exiguobacterium* sp. PMA. (**b**) Degradation of 4C2NP in non-sterile soil by *Exiguobacterium* sp. PMA, (**c**) Degradation of 4C2NP in control microcosm with sterile soil, (**d**) Degradation of 4C2NP in control microcosm with non-sterile soil.

In the test microcosm with sterile soil, there was complete removal of 4C2NP by *Exiguobacterium* sp. PMA within 12 days (Figure 5a). No degradation was observed at initial two days after incubation. On the fourth days, 10% degradation was observed and degradation was 30% by sixth days. At eight days almost 56% degradation of 4C2NP was completed. The degradation was 76% by 10 days. Almost complete degradation of 4C2NP was observed at 12 days. In another test microcosm with non-sterile soil, complete 4C2NP depletion occurred within eight days (Figure 5b). However, in controls with sterile and non sterile soils, very low degradation was observed within 12 days (Figure 5c and 5d).

## Discussion

A 4C2NP degrading bacterium, *Exiguobacterium* sp. PMA was isolated from soil collected from a contaminated site in India by an enrichment method. *Exiguobacterium* sp. PMA utilized 4C2NP as the sole carbon and energy source and degraded it up to a concentration of 0.6 mM. *Exiguobacterium* sp. PMA degraded 4C2NP with the release of stoichiometric amounts of chloride and ammonium ions.

The 4C2NP degradation pathway was studied, and 4C2AP was identified as a metabolite of the 4C2NP degradation pathway in *Exiguobacterium* sp. PMA. Literature studies showed that 4C2AP has been previously detected as an intermediate product of the degradation of various chlorinated nitroaromatic compounds [2,10,14]. Beunink and Rehm [10] reported the formation of 4C2AP in the degradation of 4C2NP, which degraded further by the release of chloride and ammonium ions. Park et al. [14] detected 4C2AP as an intermediate in the degradation pathway of 3-chloronitrobenzene, which was acetylated further to 4-chloro-2-acetaminophenol (4C2AAP). The acetylation of 4C2AP into 4C2AAP was also reported in the degradation of 4C2NP by a marine *Bacillus* sp. MW-1 [2]. In the present study, the acetylation of 4C2AP was not observed and 4C2AAP was not detected as a metabolite. These results indicate that the acetylation mechanism was not involved in the 4C2NP degradation pathway in *Exiguobacterium* sp. PMA. In the present study, 2AP was detected as another intermediate of the 4C2NP degradation pathway in *Exiguobacterium* sp. PMA, which may be formed from 4C2AP by the reductive dehalogenation. In addition, the 4C2AP dehalogenase activity was observed in the crude extract of the 4C2NP-induced PMA cells, which in turn confirmed the conversion of 4C2AP into 2AP in the 4C2NP degradation pathway in *Exiguobacterium* sp. PMA. The further degradation of 2AP proceeded by the removal of ammonium ions. This is the first report of the formation of 2AP by any bacterium in the 4C2NP degradation pathway.

**Figure 6:**
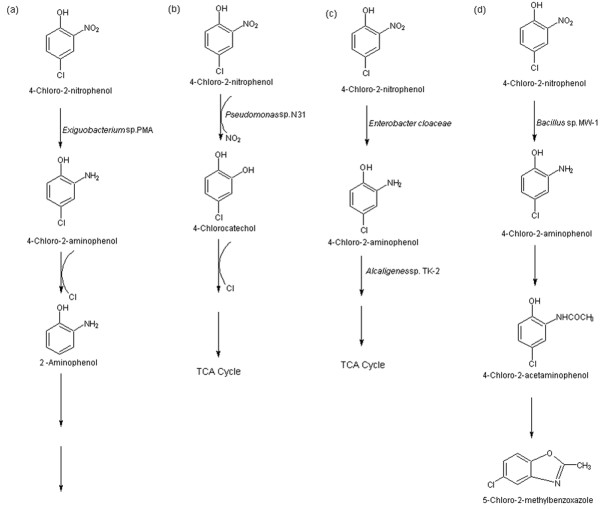
**Degradation of 4C2NP by bacteria.** (**a**) Proposed pathway of degradation of 4C2NP for *Exiguobacterium* sp. PMA. (**b**) Degradation of 4C2NP by a genetically engineered bacterium, *Pseudomonas* sp. N31. (**c**) Degradation of 4C2NP by a co culture of two bacteria. (**d**) Biotransformation of 4C2NP by *Bacillus* sp. MW-1.

On the basis of the discussion, we propose a degradation pathway of 4C2NP for *Exiguobacterium* sp. PMA (Figure 6a). Initially, 4C2NP was reduced to 4C2AP, which reductively dehalogenated to AP which was degraded further by the release of ammonium ions.

The 4C2NP degradation pathway identified in *Exiguobacterium* sp. PMA differs from previously reported degradation pathways. A genetically engineered bacterium, *Pseudomonas* sp. N31 [11] degraded 4C2NP by the oxidative removal of nitro group and formation of chlorocatechol [Figure 6b. However, *Exiguobacterium* sp. PMA degraded 4C2NP via the reductive removal of nitro group and formation of 4C2AP and 2AP. Beunink and Rehm [10] reported degradation of 4C2NP by a co-culture of two bacteria via the formation of 4C2AP and release of chloride and ammonium ions [Figure 6c. This report also differed from the present report in the formation of 2AP in the 4C2NP degradation pathway in *Exiguobacterium* sp. PMA. Another 4C2NP-degrading bacterium, *Bacillus* sp. strain MW-1 [2] biotransformed 4C2NP into 5-chloro-2-methylbenzoxazole via the formation of 4C2AP and 4C2AAP (Figure 6d). Neither 5-chloro-2-methylbenzoxazole nor 4C2AAP was detected as an intermediate in the degradation pathway of 4C2NP by *Exiguobacterium* sp. PMA.

The bioremediation potential of *Exiguobacterium* sp. PMA was investigated in soil using microcosms with 4C2NP spiked sterile and non-sterile soil. *Exiguobacterium* sp. PMA efficiently degraded 4C2NP in microcosms with sterile and non-sterile soils; however, the degradation of 4C2NP was faster in non-sterile soil than sterile soil. These data suggest that indigenous bacteria as well as biotic factors have supported the degradation of 4C2NP with *Exiguobacterium* sp. PMA. No or very less degradation was observed in non-sterile soil microcosm (control), indicated that indigenous soil bacteria have no ability to utilize 4C2NP as the sole carbon and energy source. Indigenous soil bacteria increased the rate of degradation of 4C2NP perhaps due to the utilization of intermediates that may arise in the soil due to the 4C2NP degradation by *Exiguobacterium* sp. PMA. No accumulation of any intermediate of the degradation of 4C2NP was observed in the soil. This is the first report of the 4C2NP degradation in soil. These microcosm studies will be helpful in the design of small-scale field experiments and subsequently an *in situ* 4C2NP bioremediation system for field application.

## Conclusions

*Exiguobacterium* sp. PMA utilized 4C2NP as the sole carbon and energy source and degraded it with stoichiometric release of ammonium and chloride ions. 4C2AP and 2AP were detected as two major metabolites of degradation pathway of 4C2NP in *Exiguobacterium* sp. PMA. Our studies indicate that *Exiguobacterium* sp. PMA may be useful for the bioremediation of 4C2NP-contaminated sites. This is the first report of (i) the formation of 2AP in the 4C2NP degradation pathway by any bacterium and (iii) the bioremediation of 4C2NP by any bacterium.

## Materials and methods

### Chemicals

4C2NP (97%), 4C2AP (97%), 2AP (99%), 2NP (98%), 2C4NP (97%), 2Me4NP (97%), 3Me4NP (98%) and 4NP (≥99%) were purchased from Sigma-Aldrich (GmbH, Steinheim, Germany). Methanol and ethyl acetate were procured from Merck Limited (Darmstadt, Germany). All the other chemicals used in the study were of the highest purity grade.

### Isolation of 4C2NP degrading bacteria

A 4C2NP-degrading bacterium, *Exiguobacterium* sp. PMA was isolated from soil collected from a chemically-contaminated site, Gajraula (28.85°N 78.23°E), Amroha, Uttar Pradesh, India by an enrichment method using a yellow coloured compound 4C2NP.

For enrichment, 1 g of the soil sample was added to 250 ml Erlenmeyer flask containing 100 ml minimal media and 0.2 mM 4C2NP as the sole carbon and energy source. Upon the decolourization, culture was serially diluted and plated on minimal agar plates containing 0.2 mM 4C2NP. About five different morphological colonies have selected on the basis of decolourization. All five strains have screened to their capability to degrade 4C2NP at higher concentrations (0.3-1 mM). *Exiguobacterium* sp. PMA was able to degrade 4C2NP up to a concentration of 0.6 mM and selected for further study.

*Exiguobacterium* sp. PMA was screened for its ability to degrade other nitroaromatic compounds. For screening, *Exiguobacterium* sp. PMA was streaked on minimal agar plates containing 0.3 mM test compound as the sole carbon and energy source. Minimal agar plates were prepared as described previously [10]. 2NP, 4NP, 2C4NP, 2AP, 4C2AP, 3Me4NP, and 2Me4NP were used as test compounds. Decolourization and growth of *Exiguobacterium* sp. PMA on minimal agar plates were considered positive results.

### Identification of 4C2NP degrading bacteria

*Exiguobacterium* sp. PMA was identified on the basis of the 16S rRNA gene sequencing using universal primers, 27 F (5’-AGAGTTTGATCCTGGCTCAG-3’) and 1492R (5’-TACGGYTACCTTGTTACGACTT-3’) by the method as described previously [13,15,16]. The PCR amplification reaction mix (25 μl) contained 50–100 ng of genomic DNA, 2.5 μl of 10 X Taq polymerase buffer, 200 μM of each dNTP, 1.0 U of *Taq* DNA polymerase (New England Biolabs, MA, USA), 20 pmol of each primer (BioBasic Inc. Ontario, Canada) and water. Amplification was carried out using a personal thermocycler (Eppendorf, Hamburg, Germany) [13,15,16]. Amplification program consisted of an initial denaturation at 94°C for 3 min followed by 30 cycles of denaturation at 94°C for 1 min, annealing at 55°C for 1 min, extension at 70°C for 1 min, and final extension at 72°C for 5 min [10]. The amplified PCR product was sequenced using Big Dye terminator cycle sequencing ready reaction kit (Applied Biosystems) by an automated DNA sequencer (ABI 3130 XL Genetic Analyzer; Applied Biosystems) [13,15,16]. The 16S rRNA gene sequence similarity of *Exiguobacterium* sp. PMA was determined by using BLAST.

### Minimal media and culture conditions

The minimal medium was prepared by dissolving the following compounds in 100 ml of double distilled water: 0.4 g Na_2_HPO_4_, 0.2 g KH_2_PO_4_, 0.08 g NaNO_3_, 0.08 g MgSO_4_.7H_2_O, 0.1 ml trace element solution and 1.8 g agar. The composition of trace element solution was exactly same as described previously [2]. One liter of the trace element solution contained: 0.10 g Al(OH)_3_, 0.05 g SnCl_2_·2H_2_O, 0.05 g KI, 0.05 g LiCl, 0.08 g MgSO_4_, 0.05 g H_3_BO_3_, 0.10 g ZnSO_4_·7H_2_O, 0.01 g CoCl_2_, 0.01 g NiSO_4_·6H_2_O, 0.05 g BaCl_2_, 0.05 g (NH_4_)_6_Mo_7_O_24_**·**4H_2_O [2]. *Exiguobacterium* sp. PMA was grown on minimal medium containing 0.5 mM 4C2NP at 30°C under 200 rpm.

### Growth and degradation studies

*Exiguobacterium* sp. PMA was grown in minimal medium containing 0.5 mM 4C2NP as the sole carbon and energy source. The growth of *Exiguobacterium* sp. PMA was monitored with measurement of optical density at 600 nm (OD_600_) and the degradation of 4C2NP was observed with decrease the absorbance at 420 nm. Chloride ions were analyzed using QuantiChrom™ Chloride assay kit (DICL-250) from BioAssay Systems, Hayward, CA. Ammonia ions were detected with the ’Ammonia Assay Kit’ from Sigma-Aldrich (GmbH, Germany) according to the manufacturer’s instructions.

### Effect of various substrate concentrations on 4C2NP degradation

To study the effect of initial 4C2NP concentration, *Exiguobacterium* sp. PMA was grown on minimal media containing desired concentration of 4C2NP (0.2 mM, 0.3 mM, 0.4 mM, 0.5 mM, 0.6 mM and 0.7 mM). Samples were collected at regular intervals. Degradation studies were performed as described above.

### Effect of different inoculum sizes on 4C2NP degradation

*Exiguobacterium* sp. PMA was grown on 250 ml nutrient broth at 30°C under shaking condition. When the culture reached the late logarithmic phase of growth, usually in 30 to 32 h, the cells were harvested by centrifugation at 10000 × g for 20 min at 4°C, washed with minimal medium. The resultant pellets were re-suspended in double distilled water. To study effect of different inoculum sizes on degradation, different quantities of cells suspension were added to 200 ml minimal media containing 0.5 mM 4C2NP as a sole source of carbon and energy. At different time intervals, the 4C2NP degradation was monitored. The final concentrations of the inoculum used in this study were: 3.0 × 10^6^, 3 × 10^7^, and 3 × 10^8^ CFU/ml which were confirmed at the start of the experiment by plate count method.

### Identification of metabolites

To identify the metabolites of the degradation pathway of 4C2NP, *Exiguobacterium* sp. PMA was grown on minimal media containing 0.5 mM 4C2NP; samples were collected at regular intervals (0 h, 12 h, 24 h, 36 h, 44 h) and centrifuged. The supernatant was extracted with ethylacetate and the extracted samples were analyzed by high performance liquid chromatography (HPLC) and gas chromatography–mass spectrometry (GC-MS) as described previously [2,13].

HPLC analysis was performed using a Waters 600 model HPLC equipped with a photodiode array detector system [2,11]. The 2C4NP and their metabolites were separated on a C_18_ reverse-phase silica column using 1% glacial acetic acid in methanol and 1% glacial acetic acid in HPLC grade water at a ratio of 80:20 as the mobile phase [2,13]. Flow rate was 1.0 ml/min; injection volume was 15 μl, and the compounds were detected at 280 nm and 300 nm [2].

GC-MS analysis was carried out using a GC-MS-QP5000 instrument (Shimadzu, Tokyo, Japan) equipped with quadrupole mass filter and DB-1 capillary column with ionization of 70 eV and scan interval 1.5 s [2,13]. The column temperature was initially increased from 80°C to 160°C at the rate of 5°C min^-1^ and then from 160°C to 260°C at the rate of 10°C min^-1^[2,13]. The carrier gas (Nitrogen) flow rate was 20 ml min^-1^[2,13].

### Enzyme assays with cell free lysate

In order to further strengthen the results of biochemical characterization of the 4C2NP degradation and demonstrate the induction of enzymes involved, different enzymatic assays were performed with the induced cells of *Exiguobacterium* sp. PMA.

The activity for 4C2NP nitroredutase was determined by the detection of product after incubation of reaction mixture at 30°C for 10 minutes. The reaction mixture contained 0.5 μmol 4C2NP, 0.6 μmol NADPH, 50 μmol phosphate buffer (pH-7.4) and 50–100 mg cell extract in a final volume of 1 ml. The reaction mixture without crude extract was used as a control. After 10 minutes, the sample was centrifuged and extracted with ethyl acetate. The extracted sample was analyzed by GC-MS. Crude extract was prepared as described previously [13].

The activity for 4C2AP dehalogenase was determined as the total chloride released at 30°C in a reaction contained 100 mM Tris–Acetate buffer (pH 7.5), 0.2 mM NADPH, 5–10 mg of cell-free lysate, and 200 μM of 4C2AP. The final volume of the reaction mixture was 5 ml. The reaction mixture without crude extract was used as a control. Samples were collected at regular intervals and assayed for chloride ions. Standard curve was prepared using NaCl as standard to quantify the chloride ions. Samples were also analyzed by GC-MS to detect the product of the reaction.

### Ring cleavage inhibition studies

To identify the ring cleavage substrate and quantify the metabolites formed during the degradation of 4C2NP using *Exiguobacterium* sp. PMA we have carried out inhibition studies using iron chelator viz., 2,2’-dipyridyl. Dipyridyl blocks the ring cleavage by chelating the ferrous ions required by dioxygenase for ring cleavage and as a result, terminal aromatic compound (substrate of the ring cleavage) along with other metabolites have accumulated in the media [13].

For inhibition studies, *Exiguobacterium* sp. PMA was inoculated in 200 ml Erlenmeyer flask containing 100 ml minimal media, 0.5 mM 4C2NP, 10 mM glucose and 1.5 mM 2,2’-dipyridyl. Culture samples were collected at regular intervals, centrifuged and supernatants were extracted with ethyl acetate. The extracted samples were analyzed with HPLC as described above.

### Microcosm studies

Soil used in microcosm studies was collected from outside the campus. The soil contained 37% clay, 28% silt, 35% sand, 0.16% organic carbon, 2.5 ppm phosphorus, 120 ppm potassium, 59 ppm nitrogen and had a pH of 8.8. The pH of the soil was adjusted to 7.0. Microcosms studies were performed as described previously [10]. Microcosms were prepared using 250 ml glass beakers and each backer contained 50 g of soil spiked with 100 ppm 4C2NP. Four types of microcosms were prepared (a) test microcosm with sterile soil, (b) test microcosms with non-sterile soil, (c) control microcosm with sterile soil, and (b) control microcosm with non-sterile soil [10]. Test microcosms with non-sterile and sterile soils were inoculated with pre-grown and 4C2NP induced cells of *Exiguobacterium* sp. PMA at ~ 2 × 10^7^ cells colony-forming units (CFUs) g^−1^ soil, whereas the control microcosms with sterile and non sterile soils were left non-bioaugmented. The bioaugmentation was performed by thorough mixing of the pre-grown cells of *Exiguobacterium* sp. PMA with the soil samples. All the microcosms were covered with perforated aluminium foil and incubated at 30°C for 15 days. During the incubation period all the microcosms were sprinkled with distilled water at regular intervals to compensate the loss of water via evaporation. Soil samples were removed at regular intervals, and extracted for analysis as described previously [10].

The various factors such as inoculum size, pH, temperature, and substrate concentration, affecting 4C2NP degradation in microcosm were optimized prior to the study as described previously [13]. To optimize conditions for rapid degradation of 4C2NP in soil, the effects of different factors were monitored by varying one parameter at a time while keeping the others constant [13].

To evaluate the effect of inoculum size, the cells of *Exiguobacterium* sp. PMA , were added to 100 ppm 4C2NP spiked soil at final concentrations of 2 × 10^5^, 2 × 10^6^, 2 × 10^7^, 2 × 10^8^, 2 × 10^9^ colony forming units (CFU) g^−1^ soil. Un-inoculated soil microcosms served as the control. All containers were incubated for 15 days at 30°C, and the chemicals levels in the soil were measured at different time intervals by HPLC as described above.

To study the effect of soil pH, the un-contaminated soils were adjusted to pH of 1.5, 2.5, 3.5, 4.5, 5.5, 6.5 7.5, 8.5, 9.5, 10.5, and 11.5 using 2 N HCl or 1 N NaOH. These soils were spiked with 100 ppm 4C2NP , incubated with the cells of *Exiguobacterium* sp. PMA (2 × 10^7^ CFU g^−1^ soil) at 30°C for 15 days and analyzed for the residual levels of 4C2NP at different time intervals as described above.

To study the effect of temperature, the degradation of 4C2NP in the 100 ppm 4C2NP spkied soil by the cells of *Exiguobacterium* sp. PMA (2 × 10^7^ CFU g^−1^ soil) was analyzed at 10, 20, 30, 40, 50, and 60°C for 15 days. The concentration of 4C2NP in the soil at different time intervals was determined as described above.

To study the effect of initial 4C2NP concentration, the soils were spiked with different concentrations of 4C2NP (50, 70, 100, 140, and 210 ppm ), incubated with the cells of *Exiguobacterium* sp. PMA (2 × 10^7^ CFU g^−1^ soil) at 30°C for 15 days and analyzed for 4C2NP.

### Statistical analysis and reproducibility

All experiments were performed in triplicate. The values were expressed as mean ± SD in the figures.

## Competing interests

The authors declare that they have no competing interests.

## Authors’ contributions

PKA, ASh, ASr, BDS, VPS, RM designed and performed the experimental works. PKA and ASh prepared the manuscript. All authors read and approved the final manuscript.
